# Healthcare facility readiness and availability for hypertension and type 2 diabetes care in Puno, Peru: a cross-sectional survey of healthcare facilities

**DOI:** 10.1186/s12913-025-12327-2

**Published:** 2025-02-22

**Authors:** Katherine E. Lord, Parker K. Acevedo, Lindsay J. Underhill, Gonzalo Cuentas, Sonia Paredes, Juan C. Mendoza, Percy Herrera, Victoria B. Chou, Victor G. Dávila-Román, Stella M. Hartinger, William Checkley

**Affiliations:** 1 Division of Pulmonary and Critical Care, School of Medicine, Johns Hopkins University, 1830 E Monument St, Room 555, Baltimore, MD USA; 2 Center for Global Non-Communicable Disease Research and Training, School of Medicine, Johns Hopkins University, Baltimore, MD USA; 3 Department of Medicine, Institute for Public Health and Cardiovascular Division, Global Health Center, Washington University in St. Louis, St. Louis, MO USA; 4 Hospital Base III, EsSalud, Puno, Perú; 5 Dirección Regional de Salud, Ministerio de Salud, Puno, Perú; 6 Dirección de Prevención y Control de Enfermedades No Transmisibles, Ministerio de Salud, Raras y Huerfanas, Lima, Perú; 7 Department of International Health, Program in Global Disease Epidemiology and Control, Bloomberg School of Public Health, Johns Hopkins University, Baltimore, MD USA; 8 Facultad de Salud Pública y Administración, Universidad Peruana Cayetano Heredia, Lima, Perú

**Keywords:** Hypertension, Diabetes, Healthcare facility, Service readiness, Peru

## Abstract

**Background:**

Non-communicable diseases are a rapidly growing cause of mortality and morbidity in Peru, accounting for 73% of total deaths. There is limited research on health system readiness to manage this rising burden. Our study characterized the readiness of healthcare facilities to manage patients with hypertension and/or type 2 diabetes (T2D) across two public healthcare systems in Puno, a largely rural and low-income department in Peru.

**Methods:**

We adapted the World Health Organization Service Availability and Readiness Assessment to characterize resources and services for hypertension and T2D care. We administered the survey to personnel at healthcare facilities in Puno. Service readiness scores were calculated based on tracer items necessary for hypertension or T2D care. Facilities with ≥ 70% of tracer items were considered ready to manage each disease.

**Results:**

Between June 2022 and June 2023, we surveyed 85% (414/488) of Puno’s government-run healthcare facilities. Overall, only 28% and 29% were considered ready to manage hypertension and T2D, respectively. Despite larger annual cumulative case volumes at health posts and low-level health centers, lower-level facilities were significantly less likely to be ready to manage hypertension (OR = 0.20, 95% CI 0.11–0.36) or T2D (OR = 0.03, 95% CI 0.01–0.06). Areas of concern included an overreliance on aneroid blood pressure monitors, their infrequent calibration, and limited hemoglobin A1c and urine testing. Additionally, only 66% and 48% of providers self-reported that they could diagnose hypertension and T2D, respectively. There was also low essential medication availability including insulin (7.2%) and fixed-dose combinations (0.5%) and a scarcity of trained healthcare professionals, particularly community health workers at health posts (49.8% had none).

**Conclusions:**

Healthcare facilities were largely unprepared to manage hypertension and T2D, underscoring the critical need for health system strengthening to address disparities in non-communicable disease management across Puno.

**Supplementary Information:**

The online version contains supplementary material available at 10.1186/s12913-025-12327-2.

## Introduction

Non-communicable diseases (NCDs) are the leading cause of mortality and morbidity worldwide, resulting in 74% of deaths globally [1]. Even though 77% of overall NCD deaths occur in low- and middle-income countries (LMICs) [1], many LMIC health systems continue to prioritize infectious diseases and acute maternal and child health conditions through programs like childhood vaccination campaigns and active surveillance [2]. Indeed, previous health facility surveys have found that many LMIC health systems are ill-equipped to handle NCD care [3–11]. A barrier to successfully diagnose and treat common NCDs, such as hypertension and type-2 diabetes (T2D), is the limited availability and accessibility of primary healthcare facilities and services.

Like many LMICs, Peru faces a rapidly growing burden of NCDs [12], which account for 73% of total deaths in the country [13]. Healthcare capacity to manage NCDs also varies widely across Peru, with rural regions facing the greatest inequity in resource availability [14]. In Peru and across Latin America, there is a paucity of data about the resources and capabilities of healthcare facilities to manage NCDs, making it difficult to identify gaps and design effective strategies to improve care. We have not identified previous studies that have conducted a comprehensive survey on the readiness of healthcare facilities to manage NCDs in Peru. We aimed to characterize the service readiness of healthcare facilities to manage patients with hypertension and/or T2D across two public healthcare systems in the department of Puno, Peru. We based our survey on the World Health Organization (WHO) Service Availability and Readiness Assessment (SARA), an assessment tool developed to monitor health services using a set of tracer indicators, which are key health system inputs and outputs indicative of service coverage [15].

## Methods

### Study setting

Puno is a department in southeastern Peru located between 3,812 and 5,500 m above sea level. The capital of the department, Puno City, sits near the Bolivian border on the shores of Lake Titicaca. Puno has a population of 1,172,697 (2017 census), or about 3% of Peru’s population, and is divided into 13 provinces [16].

Puno’s population is approximately 90% indigenous, largely comprised of people with Quechua and Aymara ethnicity [16]. Most residents (89%) are employed in the informal sector [17], with many working as farmers, traders, or sellers of wool handicrafts. Puno’s population has a lower life expectancy and poorer health outcomes than other regions of Peru, likely attributed to widespread poverty and limited access to healthcare [18, 19].

The survey was administered at Ministry of Health (MINSA, for the Spanish acronym) healthcare facilities, which offer medical services via government-subsidized healthcare. The survey was also administered at Social Security healthcare facilities (EsSalud, for the Spanish acronym), which are funded via a mandatory health insurance program for all formal workers, independent workers, and cooperative members. MINSA facilities serve 52% of Puno’s population whereas EsSalud serves 12% [20]. Military, police, private, and non-governmental organization-based healthcare facilities were excluded from this study.

Healthcare facilities across Peru are categorized based on their level of care as: primary (I-1 to I-4); secondary (II-1, II-2, or II-E); or tertiary (III-1, III-2, or III-E) [21]. EsSalud uses comparable tertiary classifications with different naming conventions. All facility levels in Puno were surveyed for this study, including health posts (I-1 and I-2), health centers (I-3 and I-4), and hospitals (II-1 and II-2).

### Study design

We conducted the healthcare facility survey as formative work for the Addressing Hypertension and Diabetes through Community-Engaged Systems (ANDES) randomized controlled trial, a multi-component intervention using community health workers (CHWs) to reduce blood pressure (BP) among patients with hypertension in Puno. The survey sought to identify resources and infrastructure available for scale-up of the ANDES intervention, if it is successful.

We developed the survey for use in this study (Online Supplement) as an adaptation of SARA, a comprehensive assessment of healthcare facility capabilities and readiness to deliver services used for health system monitoring and research, globally [3, 5, 6, 10, 11, 15, 22]. SARA covers general service availability and readiness as well as service-specific readiness [15]. For the current study, we used SARA questions related to general, hypertension-, and T2D-specific service readiness, adapted by study investigators in collaboration with local MINSA partners. It includes essential medications listed by WHO and MINSA as well as components of HEARTS, the WHO’s model for cardiovascular risk management [23]. The survey was initially developed in English, translated into Spanish, and back translated into English for validation. A standard operating procedure for survey administration was also developed. We then piloted the survey and made final adjustments. We aimed to administer the survey at all 467 MINSA and 21 EsSalud facilities in Puno.

### Ethics approval and consent to participate

Our study was approved by the institutional review boards of Universidad Peruana Cayetano Heredia (IRB104372) and Johns Hopkins University (IRB00345868). Since the healthcare facility survey does not involve human subject research and does not ask physicians about their individual data, the ethics committee of record (IRB104372) did not require us to obtain informed consent to obtain healthcare facility-level data (i.e., waiver of consent). We adhered to the Declaration of Helsinki guidelines.

### Data collection

The survey was administered by hired consultants at MINSA healthcare facilities and by our research team at EsSalud healthcare facilities. Primary respondents were lead physicians, nurses, or administrators, while questions related to medications and laboratory tests were directed toward staff managing the pharmacy and laboratory, respectively. For questions on equipment and medication inventories, we directly observed supplies to verify responses when feasible. Surveys typically took twenty minutes to one hour to administer, depending on facility size. All surveys were documented on paper before entry into a Research Electronic Data Capture (REDCap) database [22, 24]. The dataset was validated by reviewing discrepancies and recontacting the facility if significant outliers or missing data were identified. Quality assurance checks were completed in a random sample of 27 facilities (5.6%). Quality assurance data was compared with preliminary data to evaluate initial survey administration and any notable changes between visits.

### Statistical analysis

Exploratory data analysis was followed by descriptive statistics calculated across all survey sections. When data was missing, it was excluded from the analysis. Using SARA methodology, service readiness for hypertension and T2D was defined across four domains: staff and guidelines; basic technologies/equipment; diagnostic facility; and essential medicines. Within each domain, we selected specific tracer items necessary for adequate disease management (Table S1), as determined in consultation with local healthcare professionals and the service readiness literature [3, 5, 6, 10, 11, 15, 22]. Disease-specific service readiness was calculated at a facility by determining if each item was available and functioning on the day of the survey. We then calculated the mean availability of tracer items in each domain and the mean availability across all domains (Online Supplement, Figure S1), resulting in disease-specific service readiness scores. These scores were converted into dichotomous variables where facilities with scores ≥ 70% were considered ready to manage the disease [3, 5, 6, 10, 11, 15, 25].

We used single variable and multivariable logistic regressions to model service readiness (for hypertension and T2D) as a function of healthcare facility level and degree of urbanization. We ran separate models for each disease. The most appropriate model to describe the data and goodness of fit was determined using likelihood ratio tests. We completed statistical analyses in R (version 4.2.1) [26]. The primary and corresponding authors had full access to the data and were responsible for data integrity and analyses.

## Results

### Healthcare facility basic characteristics

We administered the survey at 414 healthcare facilities (84.8% of all target facilities; see tables for all numerators and denominators) between June 2, 2022, and June 30, 2023. In Table S2, we present the general facility characteristics, in which 3.4% were hospitals, 24% were health centers, and 73% were health posts. Most (98%) facilities surveyed were MINSA facilities and 26% served an urban population. Among the surveyed facilities, health posts had an estimated cumulative case load volume of 155,525 adult patients per year, health centers had 330,490, and hospitals had 406,928.

### Healthcare facility infrastructure

In Table S2, we summarize healthcare facility infrastructure. Most facilities had a flushing toilet (79%) and only 1.5% had no running water available. Almost all facilities (98%) had a central electricity supply; however, 59% experienced periodic electricity service disruption, often caused by storms during the rainy season. Only 20% of facilities had access to Wi-Fi or cable internet, and most (82%) kept their records on paper.

### Human resources

We identified important differences in the number of healthcare professionals at lower versus higher-level facilities (Table S3), with a median of 0 (interquartile range 0–1) doctors and 1 (0–1) nurse at health posts, and 24 (14–53) doctors and 44 (25–79) nurses at hospitals. Healthcare workers available at health posts were nurses, nurse technicians, and midwives, with a median of 4 (3–6) total full-time staff. Pharmacists and pharmacy technicians were generally only available at hospitals, while biologists, nutritionists, and laboratory technicians were available at some health centers too. CHWs were rarely available, especially at lower-level facilities, with a median of 0 (0–1) at health posts where 49.8% had no CHWs employed.

### Equipment and supplies to manage and treat hypertension

A median of 2 (1–4) aneroid and 1 (0–1) automatic functioning BP monitors were available at healthcare facilities, but this number varied based on facility type with a median of 0 (0–1) automatic monitors at health posts and 2 (1–4) at hospitals (Table S4). While 46% of facilities had more than one BP cuff size available, this percentage was skewed by the 71% of hospitals that had different sizes; in contrast, only 40% of health posts had different sizes. Additionally, most healthcare facilities (73%) across levels had never had their monitors calibrated or checked for accuracy.

### Availability of hypertension tracer items

In Table 1, we summarize hypertension tracer items available at the surveyed healthcare facilities. Overall, while most had the doctors (55%), nurses (95%), and nurse technicians (96%) essential for hypertension care, only 8.9% of facilities, mostly in hospitals, had a hypertension specialist. Few (6.5%) had an electrocardiogram machine available. Only 63% of facilities could diagnose hypertension, even though most (80%) had national diagnosis or treatment guidelines. Respondents, particularly at lower-level facilities, often reported that, while they had national guidelines, the absence of physicians limited diagnostic capacity. Most (98%) facility pharmacies had angiotensin-converting enzyme inhibitors or angiotensin receptor blockers available, and many (70%) had calcium channel blockers; however, only 2.7% and 0.5% had diuretic or combination treatment options, respectively.

**Table 1:** Availability of hypertension tracer items at healthcare facilities in Puno, Peru

**Characteristic**	**Overall**, *N* = 414^a^	**Health post**, *N *= 301^a^	**Health center**, *N* = 99^a^	**Hospital**, *N* = 14^a^
**Staff and guidelines**				
*General and specialist doctors (full-time)*	55% (229 / 414)	39% (117 / 301)	99% (98 / 99)	100% (14 / 14)
*Nurses (FT)*	95% (395 / 414)	94% (283 / 301)	99% (98 / 99)	100% (14 / 14)
*Nurse technicians (FT)*	96% (397 / 414)	95% (286 / 301)	98% (97 / 99)	100% (14 / 14)
*Consultation with a hypertension specialist*	8.9% (37 / 414)	6.0% (18 / 301)	11% (11 / 99)	57% (8 / 14)
*Guidelines for diagnosis and treatment of hypertension*	80% (332 / 414)	76% (228 / 301)	92% (91 / 99)	93% (13 / 14)
**Basic technologies/equipment**				
*BP monitor (aneroid* + *stethoscope or automatic)*	97% (401 / 414)	96% (290 / 301)	100% (99 / 99)	86% (12 / 14)
*Electrocardiogram*	6.5% (27 / 414)	0.7% (2 / 301)	13% (13 / 99)	86% (12 / 14)
**Diagnostic facility**				
*Provide hypertension diagnosis*	63% (262 / 414)	53% (161 / 301)	88% (87 / 99)	100% (14 / 14)
**Essential medicines**				
*Calcium channel blockers (at least one of amlodipine or nifedipine)*	70% (290 / 414)	76% (230 / 301)	58% (57 / 99)	21% (3 / 14)
*Angiotensin Converting Enzyme (ACE) inhibitors or Angiotensin Receptor Blocker (ARBs) [at least one of enalapril, captopril, or losartan]*	98% (405 / 414)	97% (293 / 301)	99% (98 / 99)	100% (14 / 14)
*Diuretic (Hydrochlorothiazide [HCTZ])*	2.7% (11 / 414)	0% (0 / 301)	5.1% (5 / 99)	43% (6 / 14)
*Combination treatment (at least one of lisinopril* + *amlodipine, lisinopril* + *HCTZ, telmisartan* + *amlodipine, or telmisartan* + *HCTZ)*	0.5% (2 / 414)	0% (0 / 301)	0% (0 / 99)	14% (2 / 14)

^a^% (n / N)

### Equipment and supplies to manage and treat T2D

There was a median of 1 (1–2) glucometer available at health posts, while 2 (1–3) were available at health centers, and 3 (1–5) at hospitals (Table S5). Similarly, there were more working tape measures and adult balances available at larger, higher-level facilities.

### Availability of T2D tracer items

We summarize the availability of tracer items deemed essential for T2D management in Table 2. Few (6.3%) facilities offered a patient consultation with a diabetes specialist or endocrinologist, including 43% of hospitals. Most (74%) facilities had guidelines for diagnosing and treating T2D and some could offer a T2D diagnosis (48%); however, diagnostic test availability was variable. While 80% of healthcare facilities could perform a fasting glucose test, only 21% could measure urinary ketones. Additionally, 37% of healthcare facilities could measure urinary glucose and protein, while only 27% could measure HbA1C levels. During quality control, it was noted that while many facilities had the test strips available to measure diabetes-related markers, many technicians or nurses interviewed were unaware of all the markers the strips could measure. Only 7.2% of pharmacies had any insulin in stock. While most (78%) pharmacies could supply metformin to their patients, only 1.9% (including 0% of health posts) had gliclazide stocked.

**Table 2:** Availability of type 2 diabetes (T2D) tracer items at healthcare facilities in Puno, Peru

**Characteristic**	**Overall**, *N* = 414^a^	**Health post**, *N* = 301^a^	**Health center**, *N* = 99^a^	**Hospital**, *N* = 14^a^
**Staff and guidelines**				
*General and specialist doctors (full time [FT)]*	55% (229 / 414)	39% (117 / 301)	99% (98 / 99)	100% (14 / 14)
*Nurses (FT)*	95% (395 / 414)	94% (283 / 301)	99% (98 / 99)	100% (14 / 14)
*Nurse technicians (FT)*	96% (397 / 414)	95% (286 / 301)	98% (97 / 99)	100% (14 / 14)
*Consultation with a diabetes specialist or endocrinologist*	6.3% (26 / 414)	3.7% (11 / 301)	9.1% (9 / 99)	43% (6 / 14)
*Guidelines for diagnosis and treatment of T2D*	74% (305 / 414)	67% (203 / 301)	90% (89 / 99)	93% (13 / 14)
**Basic technologies/equipment**				
*Adult weighing scale*	100% (412 / 414)	100% (301 / 301)	99% (98 / 99)	93% (13 / 14)
*Tape measure/height board*	97% (401 / 414)	97% (291 / 301)	98% (97 / 99)	93% (13 / 14)
*Glucometer*	97% (402 / 414)	98% (294 / 301)	97% (96 / 99)	86% (12 / 14)
*BP monitor (aneroid* + *stethoscope or automatic)*	97% (401 / 414)	96% (290 / 301)	100% (99 / 99)	86% (12 / 14)
**Diagnostic facility**				
*Fasting glucose*	80% (331 / 414)	73% (221 / 301)	98% (97 / 99)	93% (13 / 14)
*Hemoglobin A1c*	27% (110 / 414)	17% (52 / 301)	49% (49 / 99)	64% (9 / 14)
*Glucose in urine*	37% (153 / 414)	22% (66 / 301)	77% (76 / 99)	79% (11 / 14)
*Protein in urine*	37% (152 / 414)	20% (60 / 301)	81% (80 / 99)	86% (12 / 14)
*Ketones in urine*	21% (88 / 414)	6.0% (18 / 301)	60% (59 / 99)	79% (11 / 14)
*Provide diabetes diagnosis*	48% (199 / 414)	33% (99 / 301)	87% (86 / 99)	100% (14 / 14)
**Essential medicines**				
*Insulin (regular, intermediate/isophane/NPH, mixed human, short-acting analogue, long-acting analogue, mixed analogue)*	7.2% (30 / 414)	2.7% (8 / 301)	12% (12 / 99)	71% (10 / 14)
*Metformin*	78% (324 / 414)	73% (220 / 301)	91% (90 / 99)	100% (14 / 14)
*Glibenclamide*	49% (202 / 414)	39% (116 / 301)	74% (73 / 99)	93% (13 / 14)
*Gliclazide*	1.9% (8 / 414)	0% (0 / 301)	4.0% (4 / 99)	29% (4 / 14)

^a^% (n / N)

### Service readiness index scores

**Fig. 1:**
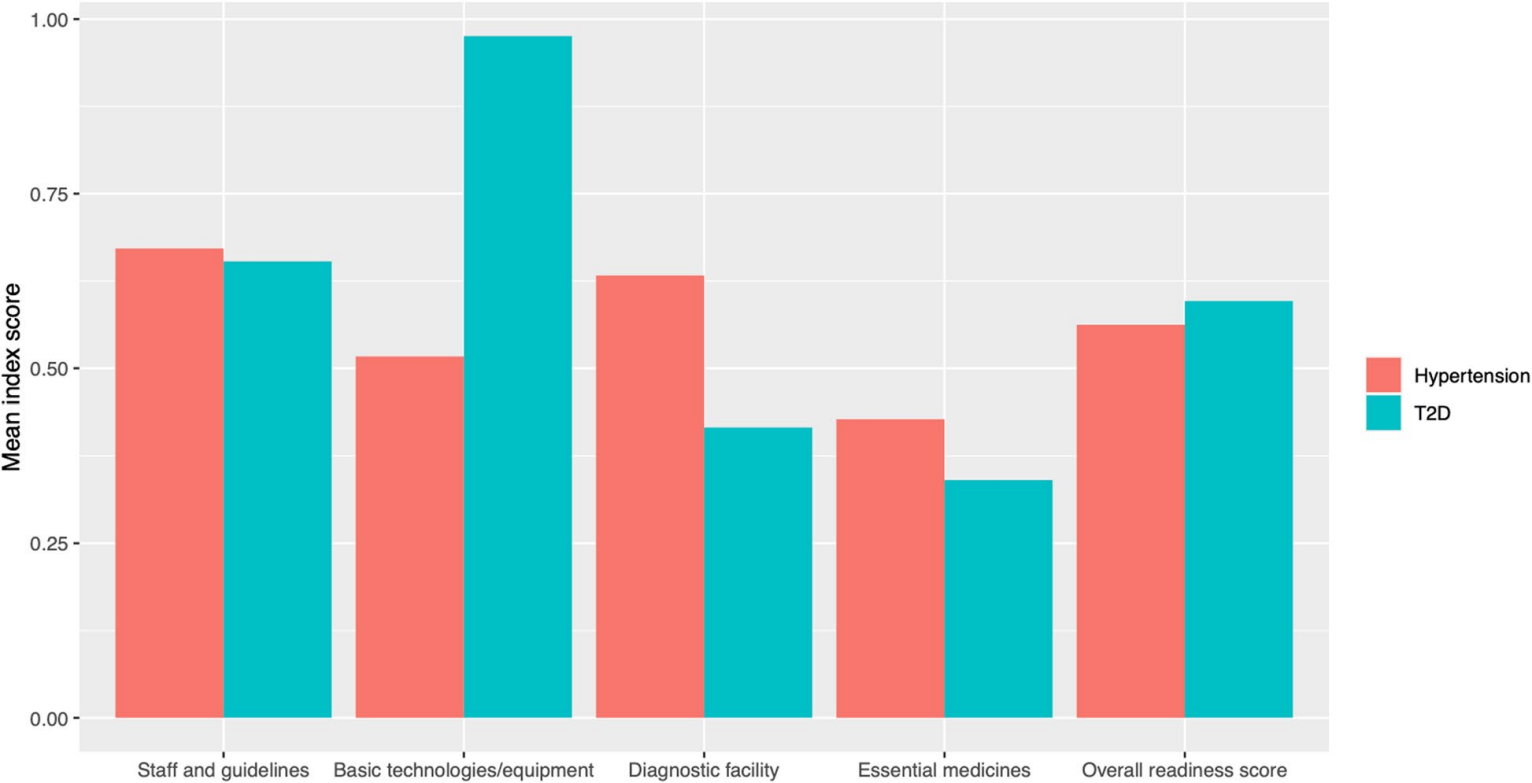
Mean tracer item index scores overall and across staff and guidelines, basic technologies/equipment, diagnostic facility, and essential medicines domains for hypertension and type 2 diabetes (T2D) care at health facilities in Puno, Peru

We summarize service readiness index scores for hypertension and T2D across healthcare facility levels in Table 3. Overall, the readiness index score for T2D was higher, with a mean (standard deviation) score of 0.60 (0.15) across facilities compared to 0.56 (0.16) for hypertension (Fig. 1). For both diseases, mean service readiness scores increased with increasing facility level.

**Table 3:** Service readiness index scores and percentage of facilities considered ready to manage type 2 diabetes (T2D) and hypertension in Puno, Peru

**Characteristic**	**Overall**, *N* = 414^a^	**Health post**, *N* = 301^a^	**Health center**, *N* = 99^a^	**Hospital**, *N* = 14^a^
**T2D**				
*Service readiness index score*	0.60 (0.15)	0.54 (0.11)	0.74 (0.10)	0.83 (0.10)
*Percentage of facilities considered ‘ready’ to manage T2D (*≥ *70% of tracer items)*	29% (120 / 414)	10.0% (30 / 301)	78% (77 / 99)	93% (13 / 14)
**Hypertension**				
*Service readiness index score*	0.56 (0.16)	0.52 (0.15)	0.66 (0.11)	0.80 (0.08)
*Percentage of facilities considered ‘ready’ to manage hypertension (*≥ *70% of tracer items)*	28% (116 / 414)	16% (48 / 301)	56% (55 / 99)	93% (13 / 14)

^a^Mean (SD)

**Fig. 2:**
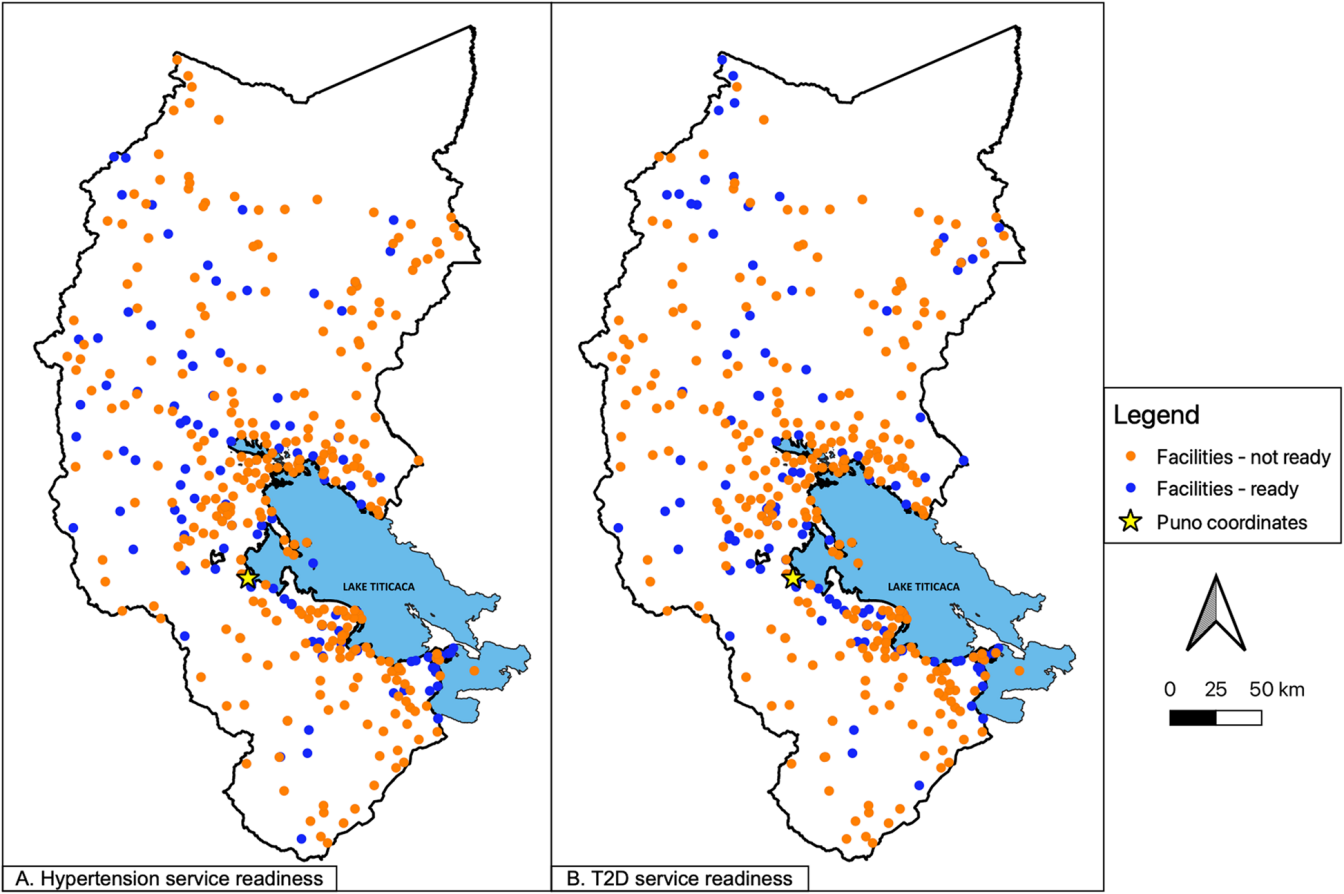
Map of Puno, Peru displaying all healthcare facilities surveyed in this study based on whether they were considered prepared to manage hypertension (**a**) or type 2 diabetes (T2D) (**b**)

We summarize the percentage of facilities at each level that reached the 70% service readiness threshold in Table 3. These results are displayed in Fig. 2, which shows that facilities considered more prepared to manage hypertension and T2D were generally located closer to Lake Titicaca and Puno City. Across facilities, 28% and 29% were considered ready to manage hypertension and T2D, respectively. The percentage of facilities reaching the 70% threshold increased as facility level increased.

### Assessing factors contributing to hypertension and T2D service readiness

Adjusting for facility level, urban facilities were 2.29 times more likely to be considered ready to manage hypertension; however, there were no significant differences in T2D service readiness between urban and rural facilities (Table 4). Facility level was also an important predictor of both T2D and hypertension service readiness, with I-1 and I-2 facilities significantly less likely to be considered ready to manage either hypertension (OR = 0.20, 95% CI 0.11–0.36) or T2D (OR = 0.03, 95% CI 0.01–0.06) compared to I-3 or higher-level facilities.

**Table 4:** Factors associated with type 2 diabetes (T2D) and hypertension service readiness at healthcare facilities in Puno, Peru

	**T2D service readiness**							**Hypertension service readiness**						
	**Unadjusted**				**Adjusted**			**Unadjusted**				**Adjusted**		
**Characteristic**	**N**	**OR**^a^	**95% CI**^a^	***p*-value**	**OR**^a^	**95% CI**^a^	***p*-value**	**N**	**OR**^a^	**95% CI**^a^	***p*-value**	**OR**^a^	**95% CI**^a^	***p*-value**
Urban facility setting (with rural as reference)	414	8.59	5.29, 14.2	< 0.001	1.33	0.60, 2.77	0.50	414	5.78	3.60, 9.37	< 0.001	2.29	1.24, 4.15	0.007
Lower facility level (< I-3) (with ≥I-3 facilities as reference)	414	0.03	0.01, 0.05	< 0.001	0.03	0.01, 0.06	< 0.001	414	0.13	0.08, 0.20	< 0.001	0.20	0.11, 0.36	< 0.001

^a^*OR* Odds ratio, *CI* Confidence interval

## Discussion

We found that healthcare facilities in Puno had low mean readiness scores for both hypertension and T2D, indicating that most facilities were unprepared to manage these NCDs. Rural centers were less prepared, identifying an important inequity. Higher level healthcare facilities were better resourced than lower level ones, also highlighting a critical gap as health posts and centers together had a higher patient volume then hospitals. T2D readiness scores were higher than hypertension scores at every healthcare facility level. However, this was largely influenced by the high availability of T2D equipment tracer items, in comparison to hypertension which had low electrocardiogram availability.

Our survey revealed a lack of essential medications across healthcare facilities, poor training for available hypertension and T2D equipment, and a scarcity of healthcare professionals, particularly hypertension and T2D specialists. While the relative absence of specialists at health posts was expected given these facilities focus on providing primary care, the low specialist presence at health centers (specialist consultation available for hypertension and diabetes at 11% and 9.1% of facilities, respectively) and hospitals (specialist consultation available for hypertension and diabetes at 57% and 43% of facilities, respectively) highlights the need for a paradigm shift to train and recruit physicians to manage NCDs in settings like Puno. CHWs were also rarely employed, despite their importance in task shifting in rural and low resource settings [27]. Among surveyed facilities, only 41% had a supply of electricity without frequent or intermittent interruptions, and this percentage was between 44% and 67% in hospitals. There was also more aneroid BP monitors in use and limited cuff size availability or regular calibration, despite evidence to support the use of automatic cuffs [28], correct cuff size [29], and calibration every six months [30] for accurate BP measurements. Essential tests for T2D care were also limited: while fasting glucose tests were common, hemoglobin A1c and urine tests were all found, on average, at less than 50% of facilities, including in 22% or less of health posts. Quality assurance visits revealed that urine test strips were often found in facilities where the personnel interviewed had reported that the facility could not perform the tests. There was also limited availability of information technology for health data management and a reliance on manual records, with 82% of healthcare facilities relying exclusively on paper records and only 20% having access to Wi-Fi or internet.

Health facility surveys in other LMICs have also shown limited service readiness and inadequate preparation to manage NCDs [3–11], with several finding facility level and urbanization important for service readiness. A 2022 diabetes service readiness study in Bangladesh found that only tertiary facilities were ready to manage diabetes [3], similar to a 2021 study in Bangladesh which found that rural settings had the lowest capacity to manage diabetes [5]. Similarly, a 2022 study in Kenya [6] and a 2018 study in Zambia [11] found that, in general, the likelihood of being ready to manage NCDs was greater in urban and higher-level healthcare facilities.

A low stock of insulin at healthcare facilities in Puno contrasts with reports from a 2018 study which found > 60% availability of regular and NPH insulin [8]. However, this study was not conducted in Puno, one of the most resource-poor regions of Peru [19]. Like the 2018 study, our survey also found greater access to insulin at higher-level healthcare facilities. The low availability of essential T2D medications, particularly insulin and gliclazide, was also found in a 2021 survey conducted in Tanzania [9]. Overall, none of the T2D or hypertension medications (other than angiotensin converting enzyme inhibitors or angiotensin receptor blockers) were found at the recommended WHO Global Action Plan for the Prevention and Control of NCDs target of 80% availability [31], indicating poor preparation to manage these diseases in Puno. Low stocks of anti-hypertensives and anti-hyperglycemic medications in Puno may also be affected by non-centralized mechanisms for acquiring medicines and technologies [32].

A major strength of the survey was the domain comprehensiveness and the inclusion of questions for both hypertension and T2D. Another strength was the large sample size, capturing approximately 85% of MINSA and EsSalud facilities in Puno. The study also has some limitations. Selection bias may have been introduced if data which could not be collected in certain regions was systematically different from collected data. Also, due to limited literature on weighting tracer items, each item was equally weighted during index construction. Since some tracer items might be more important for service delivery than others, overall scores could be under or overestimated. Additionally, the service readiness scores did not take into consideration the infrastructure or broad-level facility or system processes necessary to effectively manage hypertension or T2D. Since the survey was only administered once, it is not possible to assess changes over time. Our survey also did not assess how well-equipped healthcare facilities were to manage patients from different cultural backgrounds (e.g., individuals who only speak Aymara or Quechua) and how these factors would affect service readiness and care access. Information bias due to self-reported data was also possible if the respondent answered incorrectly due to recall challenges or to make the facility appear more resourced than it was. This could result in an overestimation of service readiness. To limit this effect, we conducted quality assurance checks and, whenever possible, verified the availability of medicines or functioning equipment by visually checking and counting inventories at the facility.

Our analysis has identified shortcomings in Puno’s health system, contributing to their inequity in health outcomes compared to the rest of Peru. Many of the identified challenges could be quickly addressed by MINSA and EsSalud, particularly as the healthcare system implements the HEARTS initiative, an internationally standardized strategy for cardiovascular risk management. Nationally, HEARTS was set to begin in September 2019, however, due to the COVID-19 pandemic and political instability in 2022–2023, the initiative was not officially approved until May 2024. Our research was conducted before this date which offers the opportunity for a natural experiment: specifically, we could repeat the same survey across all public healthcare facilities to measure the impact of HEARTS on hypertension and diabetes management in Puno. Beyond opportunities for future research, our current results also indicate natural solutions to the identified challenges. For example, increasing the availability of functioning automatic BP monitors and different cuff sizes would improve capacity to diagnose and monitor hypertension. The government could implement a supplementary training program to increase awareness of the use of urine dipstick tests to manage T2D. Additional funding should also be funneled into task shifting, training CHWs to provide the essential, basic services of a strong NCD management program, such as accurately measuring BP and providing culturally sensitive lifestyle education. MINSA and EsSalud should also put significant focus on increasing the accessibility of information technology at their healthcare facilities, particularly through electronic medical records and Wi-Fi. This would improve the sharing of data and referral systems between facilities, particularly if a patient initially seen at a lower-level facility is referred to a hospital or vice versa, as regularly happens in NCD care. In general, MINSA and EsSalud should focus on improving ancillary services at health posts and centers since these facilities were considered more unprepared to manage hypertension and T2D despite serving a higher volume of patients. If addressed rapidly and efficiently, these recommendations could represent cost-effective measures to mitigate existing disparities in Puno.

## Conclusions

We identified significant gaps in NCD service readiness in Puno, aligning with the challenges experienced by health systems addressing NCD care in other LMICs. To our knowledge, this study is one of the first evaluating readiness to provide hypertension and T2D care in Latin America and highlights the urgent need for continued research to support health system strengthening across the region. Based on our results, healthcare administrators in Puno (and more widely throughout Peru) should prioritize improving the availability of automatic blood pressure monitors, providing training on the use of urine dipstick tests, increasing the accessibility of information technology at facilities, and training community health workers to address NCD care. Implementing these recommendations would also decrease inequities in access to quality healthcare across Puno and other departments in Peru. While our conclusions might not be externally valid to better resourced areas in Peru, many of our recommendations could be applied to other, under resourced regions of Peru and Latin America. This survey could also be used to assess the health systems of other Peruvian departments, providing important points of comparison. By improving the capacity for hypertension and T2D care across healthcare facility levels, the burden of NCD-related morbidity and mortality could be significantly reduced in Puno.

## Supplementary Information

Supplementary Material 1.

## Data Availability

Data is provided with the manuscript and supplementary files. Any additional from the datasets used and/or analysed during the current study are available from the corresponding author on reasonable request.

## Abbreviations

ANDES: Addressing Hypertension and Diabetes through Community-Engaged Systems
BP: Blood pressure
CHW: Community health worker
EsSalud: Peruvian Social Security healthcare system (acronym in Spanish)
LMIC: Low- and middle-income country
MINSA: Peruvian Ministry of Health (acronym in Spanish)
NCD: Non-communicable disease
OR: Odds ratio
SARA: Service Availability and Readiness Assessment
T2D: Type-2 diabetes
WHO: World Health Organization
