# Supplementary material for: Healthcare facility readiness and availability for hypertension and type 2 diabetes care in Puno, Peru: a cross-sectional survey of healthcare facilities
Source: BMC Health Serv Res. 2025 Feb 22;25:297. doi: 10.1186/s12913-025-12327-2 (PMC11846368; doi:10.1186/s12913-025-12327-2)
Supplement: Supplementary file 1 — Supplementary Material 1. [file 12913_2025_12327_MOESM1_ESM.docx]

**Online Supplement for “Healthcare facility readiness and availability for hypertension and type 2 diabetes care in Puno, Peru: a cross-sectional survey of health care facilities”**

Table of Contents

[Authors 2](#_Toc170399044)

[Figure S1. The approach for calculating mean availability of tracer items for hypertension and T2D. 3](#_Toc170399045)

[Table S1. Akaike Information Criterion (AIC) comparisons for selecting the best model to predict hypertension and T2D service readiness at healthcare facilities in Puno, Peru. 4](#_Toc170399046)

[Table S2. Site characteristics of healthcare facilities in Puno, Peru (extended) 5](#_Toc170399047)

[Table S3. Availability of blood pressure measurement tools at healthcare facilities in Puno, Peru 8](#_Toc170399048)

[Table S4. Availability of T2D measurement tools at healthcare facilities in Puno, Peru 9](#_Toc170399049)

[STROBE Checklist 10](#_Toc170399050)

# **Authors**

Katherine E. Lord, Parker K. Acevedo, Lindsay J. Underhill, Gonzalo Cuentas, Sonia Paredes, Juan C Mendoza, Percy Herrera, Victoria B. Chou, Victor G. Dávila-Román, Stella M. Hartinger, William Checkley

# **Figure S1.** The approach for calculating mean availability of tracer items for hypertension and T2D.

1) Determining if each tracer item was available, in which if the facility had the tracer item available and functioning (if applicable) on the day of the survey it was counted as ‘available’.

2) Calculating the mean availability of tracer items in each individual domain:

$$\frac{number of tracer items available}{all possible tracer items in that domain}\times100\%$$

3) Calculating the mean availability of tracer items across all domains:

$$\frac{\begin{aligned} percentage of staff and guideline tracer items available+percentage of basic technology & equipment tracer items available \\ + percentage of diagnostic facility tracer items available+percentage of essential medicine tracer items available \end{aligned}}{4}$$

**Table S1. Akaike Information Criterion (AIC) comparisons for selecting the best model to predict hypertension and T2D service readiness at healthcare facilities in Puno, Peru.** We calculated AIC scores for each of the models to evaluate how well each model fit the data. The quality of each model compared to other possible models with different predictors was determined in terms of prediction for hypertension and T2D service readiness, and the models with the lowest AIC scores were selected. We also used likelihood ratio tests to determine goodness-of-fit between each model and its null comparator without the added covariates. For both hypertension and T2D service readiness, we chose the model with the urbanization, health facility level, and province covariates.

| **Model** | **AIC score** |
| --- | --- |
| **Hypertension service readiness** |  |
| *htn_index_b ~ s1_urban* | 441.41 |
| *htn_index_b ~ s1_nivel_de_salud* | 375.08 |
| *htn_index_b ~ s1_provincia* | 480.62 |
| *htn_index_b ~ s1_urban + s1_nivel_de_salud* | 373.71 |
| *htn_index_b ~ s1_urban + s1_nivel_de_salud + s1_provincia* | 364.21 |
| **T2D service readiness** |  |
| *diab_index_b ~ s1_urban* | 422.67 |
| *diab_index_b ~ s1_nivel_de_salud* | 279.98 |
| *diab_index_b ~ s1_provincia* | 477.84 |
| *diab_index_b ~ s1_urban + s1_nivel_de_salud* | 281.97 |
| *diab_index_b ~ s1_urban + s1_nivel_de_salud + s1_provincia* | 250.75 |

# **Table S2. Site characteristics of healthcare facilities in Puno, Peru (extended)**

| **Characteristic**^1^ | **Overall**, N = 414 | **Health post**, N = 301 | **Health center**, N = 99 | **Hospital**, N = 14 |
| --- | --- | --- | --- | --- |
| **Service provider, % (num/den)** |  |  |  |  |
| *EsSalud* | 2.2% (9 / 414) | 0.0% (0 / 301) | 6.1% (6 / 99) | 21.4% (3 / 14) |
| *MINSA* | 97.8% (405 / 414) | 100.0% (301 / 301) | 93.9% (93 / 99) | 78.6% (11 / 14) |
| **Total healthcare staff (full-time), median (interquartile range)** | 5 (4, 11) | 4 (3, 6) | 19 (11, 25) | 162 (99, 293) |
| **Number of adult patients seen yearly, median (interquartile range)** | 510 (216, 1,114) | 370 (181, 615) | 1,800 (986, 3,664) | 10,268 (5,724, 23,375) |
| **Open 24/7, % (num/den)** | 8.2% (34 / 414) | 0.7% (2 / 301) | 20.2% (20 / 99) | 85.7% (12 / 14) |
| **Urban, % (num/den)** | 26.3% (109 / 414) | 9.3% (28 / 301) | 67.7% (67 / 99) | 100.0% (14 / 14) |
| **Primary source of water, % (num/den)** |  |  |  |  |
| *Bottled water* | 0.2% (1 / 410) | 0.3% (1 / 298) | 0.0% (0 / 98) | 0.0% (0 / 14) |
| *Cart with tank or small can* | 0.2% (1 / 410) | 0.3% (1 / 298) | 0.0% (0 / 98) | 0.0% (0 / 14) |
| *Collected rainwater* | 0.5% (2 / 410) | 0.7% (2 / 298) | 0.0% (0 / 98) | 0.0% (0 / 14) |
| *Drilled or tapped well* | 3.4% (14 / 410) | 4.4% (13 / 298) | 1.0% (1 / 98) | 0.0% (0 / 14) |
| *No source of water* | 1.5% (6 / 410) | 1.7% (5 / 298) | 1.0% (1 / 98) | 0.0% (0 / 14) |
| *Other* | 0.2% (1 / 410) | 0.3% (1 / 298) | 0.0% (0 / 98) | 0.0% (0 / 14) |
| *Pipe inside the establishment* | 58.8% (241 / 410) | 48.0% (143 / 298) | 86.7% (85 / 98) | 92.9% (13 / 14) |
| *Pipe inside the premises of the establishment* | 19.8% (81 / 410) | 24.5% (73 / 298) | 7.1% (7 / 98) | 7.1% (1 / 14) |
| *Protected spring* | 1.7% (7 / 410) | 2.3% (7 / 298) | 0.0% (0 / 98) | 0.0% (0 / 14) |
| *Protected well* | 6.3% (26 / 410) | 8.7% (26 / 298) | 0.0% (0 / 98) | 0.0% (0 / 14) |
| *Public faucet or tap* | 1.5% (6 / 410) | 1.7% (5 / 298) | 1.0% (1 / 98) | 0.0% (0 / 14) |
| *Surface water* | 0.2% (1 / 410) | 0.3% (1 / 298) | 0.0% (0 / 98) | 0.0% (0 / 14) |
| *Unprotected spring* | 0.7% (3 / 410) | 1.0% (3 / 298) | 0.0% (0 / 98) | 0.0% (0 / 14) |
| *Unprotected well* | 4.1% (17 / 410) | 5.0% (15 / 298) | 2.0% (2 / 98) | 0.0% (0 / 14) |
| *Water truck* | 0.7% (3 / 410) | 0.7% (2 / 298) | 1.0% (1 / 98) | 0.0% (0 / 14) |
| **Type of toilet facility, % (num/den)** |  |  |  |  |
| *Composting latrine* | 1.2% (5 / 406) | 1.7% (5 / 295) | 0.0% (0 / 97) | 0.0% (0 / 14) |
| *Flushing toilet* | 79.1% (321 / 406) | 72.2% (213 / 295) | 96.9% (94 / 97) | 100.0% (14 / 14) |
| *Hanging toilet or latrine* | 1.2% (5 / 406) | 1.7% (5 / 295) | 0.0% (0 / 97) | 0.0% (0 / 14) |
| *No facility, the bush, or an open field* | 3.2% (13 / 406) | 4.1% (12 / 295) | 1.0% (1 / 97) | 0.0% (0 / 14) |
| *Pit latrine with a slab* | 3.9% (16 / 406) | 5.4% (16 / 295) | 0.0% (0 / 97) | 0.0% (0 / 14) |
| *Pit latrine without a slab / open pit* | 4.2% (17 / 406) | 5.8% (17 / 295) | 0.0% (0 / 97) | 0.0% (0 / 14) |
| *Ventilated pit latrine* | 7.1% (29 / 406) | 9.2% (27 / 295) | 2.1% (2 / 97) | 0.0% (0 / 14) |
| **Primary source of electricity, % (num/den)** |  |  |  |  |
| *Central electricity supply* | 97.8% (399 / 408) | 97.0% (287 / 296) | 100.0% (98 / 98) | 100.0% (14 / 14) |
| *Generator* | 0.5% (2 / 408) | 0.7% (2 / 296) | 0.0% (0 / 98) | 0.0% (0 / 14) |
| *Other* | 0.2% (1 / 408) | 0.3% (1 / 296) | 0.0% (0 / 98) | 0.0% (0 / 14) |
| *Solar energy* | 1.5% (6 / 408) | 2.0% (6 / 296) | 0.0% (0 / 98) | 0.0% (0 / 14) |
| **Electricity interruptions, % (num/den)** |  |  |  |  |
| *Frequent/occasional interruptions* | 59.5% (242 / 407) | 60.0% (177 / 295) | 58.2% (57 / 98) | 57.1% (8 / 14) |
| *No interruptions* | 40.5% (165 / 407) | 40.0% (118 / 295) | 41.8% (41 / 98) | 42.9% (6 / 14) |
| **Access to Wi-Fi or internet, % (num/den)** | 19.8% (82 / 414) | 6.3% (19 / 301) | 49.5% (49 / 99) | 100.0% (14 / 14) |
| **Record-keeping, % (num/den)** |  |  |  |  |
| *Both computer and paper systems* | 16.2% (63 / 389) | 14.0% (40 / 286) | 18.9% (17 / 90) | 46.2% (6 / 13) |
| *Computer system only* | 1.8% (7 / 389) | 0.0% (0 / 286) | 5.6% (5 / 90) | 15.4% (2 / 13) |
| *Paper system only* | 82.0% (319 / 389) | 86.0% (246 / 286) | 75.6% (68 / 90) | 38.5% (5 / 13) |
| **Pharmacy in facility, % (num/den)** | 99.3% (411 / 414) | 99.7% (300/ 301) | 98.0% (97 / 99) | 100.0% (14 / 14) |
| ^1^Missing values were excluded from summary measure calculation | | | | |

# **Table S3. Availability of blood pressure measurement tools at healthcare facilities in Puno, Peru**

| **Characteristic** | **Overall**, N = 414^1^ | **Health post**, N = 301^1^ | **Health center**, N = 99^1^ | **Hospital**, N = 14^1^ |
| --- | --- | --- | --- | --- |
| **Number of working aneroid BP cuffs available** | 2 (1, 4) | 2 (1, 3) | 3 (2, 4) | 6 (4, 17) |
| **Number of working automatic BP cuffs available** | 1 (0, 1) | 0 (0, 1) | 1 (0, 2) | 2 (1, 4) |
| **Different cuff sizes available** | 46% (191 / 414) | 40% (120 / 301) | 62% (61 / 99) | 71% (10 / 14) |
| **Frequency of BP cuff calibration (if available)** |  |  |  |  |
| *Annually* | 6.3% (26 / 411) | 6.0% (18 / 299) | 6.1% (6 / 99) | 15% (2 / 13) |
| *Every 6 months* | 5.8% (24 / 411) | 5.7% (17 / 299) | 6.1% (6 / 99) | 7.7% (1 / 13) |
| *Every month or more* | 1.9% (8 / 411) | 0.3% (1 / 299) | 5.1% (5 / 99) | 15% (2 / 13) |
| *Less than once a year* | 12% (51 / 411) | 13% (38 / 299) | 12% (12 / 99) | 7.7% (1 / 13) |
| *Never* | 73% (302 / 411) | 75% (225 / 299) | 71% (70 / 99) | 54% (7 / 13) |

# **Table S4. Availability of T2D measurement tools at healthcare facilities in Puno, Peru**

| **Characteristic** | **Overall**, N = 414^1^ | **Health post**, N = 301^1^ | **Health center**, N = 99^1^ | **Hospital**, N = 14^1^ |
| --- | --- | --- | --- | --- |
| **Number of working glucometers available** | 1 (1, 2) | 1 (1, 2) | 2 (1, 3) | 3 (1, 5) |
| **Number of working tape measures available** | 2 (1, 3) | 2 (1, 3) | 3 (2, 4) | 8 (6, 10) |
| **Number of working adult balances available** | 2 (1, 4) | 2 (1, 3) | 3 (2, 5) | 8 (6, 10) |

# **STROBE Checklist**

STROBE Statement—Checklist of items that should be included in reports of ***cross-sectional studies***

|  | Item No | Recommendation | Page No |
| --- | --- | --- | --- |
| **Title and abstract** | 1 | (*a*) Indicate the study’s design with a commonly used term in the title or the abstract | 2 |
|  |  | (*b*) Provide in the abstract an informative and balanced summary of what was done and what was found | 2 |
| Introduction | | | |
| Background/rationale | 2 | Explain the scientific background and rationale for the investigation being reported | 4 |
| Objectives | 3 | State specific objectives, including any prespecified hypotheses | 4 |
| Methods | | | |
| Study design | 4 | Present key elements of study design early in the paper | 4­–5 |
| Setting | 5 | Describe the setting, locations, and relevant dates, including periods of recruitment, exposure, follow-up, and data collection | 4–6 |
| Participants | 6 | (*a*) Give the eligibility criteria, and the sources and methods of selection of participants | N/A |
| Variables | 7 | Clearly define all outcomes, exposures, predictors, potential confounders, and effect modifiers. Give diagnostic criteria, if applicable | 4­–5 |
| Data sources/ measurement | 8* | For each variable of interest, give sources of data and details of methods of assessment (measurement). Describe comparability of assessment methods if there is more than one group | 4­–5 |
| Bias | 9 | Describe any efforts to address potential sources of bias | 5 |
| Study size | 10 | Explain how the study size was arrived at | 5 |
| Quantitative variables | 11 | Explain how quantitative variables were handled in the analyses. If applicable, describe which groupings were chosen and why | 5 |
| Statistical methods | 12 | (*a*) Describe all statistical methods, including those used to control for confounding | 5 |
|  |  | (*b*) Describe any methods used to examine subgroups and interactions | 5 |
|  |  | (*c*) Explain how missing data were addressed | 5 |
|  |  | (*d*) If applicable, describe analytical methods taking account of sampling strategy | N/A |
|  |  | (*e*) Describe any sensitivity analyses | N/A |
| Results | | | |
| Participants | 13* | (a) Report numbers of individuals at each stage of study—eg numbers potentially eligible, examined for eligibility, confirmed eligible, included in the study, completing follow-up, and analysed | N/A |
|  |  | (b) Give reasons for non-participation at each stage | N/A |
|  |  | (c) Consider use of a flow diagram | N/A |
| Descriptive data | 14* | (a) Give characteristics of study participants (eg demographic, clinical, social) and information on exposures and potential confounders | 6–7 |
|  |  | (b) Indicate number of participants with missing data for each variable of interest | 14–20 |
| Outcome data | 15* | Report numbers of outcome events or summary measures | 14–20 |
| Main results | 16 | (*a*) Give unadjusted estimates and, if applicable, confounder-adjusted estimates and their precision (eg, 95% confidence interval). Make clear which confounders were adjusted for and why they were included | 6–7, 21 |
|  |  | (*b*) Report category boundaries when continuous variables were categorized | 5 |
|  |  | (*c*) If relevant, consider translating estimates of relative risk into absolute risk for a meaningful time period | N/A |
| Other analyses | 17 | Report other analyses done—eg analyses of subgroups and interactions, and sensitivity analyses | N/A |
| Discussion | | | |
| Key results | 18 | Summarise key results with reference to study objectives | 7 |
| Limitations | 19 | Discuss limitations of the study, taking into account sources of potential bias or imprecision. Discuss both direction and magnitude of any potential bias | 8 |
| Interpretation | 20 | Give a cautious overall interpretation of results considering objectives, limitations, multiplicity of analyses, results from similar studies, and other relevant evidence | 7–9 |
| Generalisability | 21 | Discuss the generalisability (external validity) of the study results | 9 |
| Other information | | | |
| Funding | 22 | Give the source of funding and the role of the funders for the present study and, if applicable, for the original study on which the present article is based | 9 |

*Give information separately for exposed and unexposed groups.

**Note:** An Explanation and Elaboration article discusses each checklist item and gives methodological background and published examples of transparent reporting. The STROBE checklist is best used in conjunction with this article (freely available on the Web sites of PLoS Medicine at http://www.plosmedicine.org/, Annals of Internal Medicine at http://www.annals.org/, and Epidemiology at http://www.epidem.com/). Information on the STROBE Initiative is available at www.strobe-statement.org.
